# BDNF Actions in the Cardiovascular System: Roles in Development, Adulthood and Response to Injury

**DOI:** 10.3389/fphys.2019.00455

**Published:** 2019-04-26

**Authors:** Pouneh Kermani, Barbara Hempstead

**Affiliations:** ^1^Department of Medicine, Weill Cornell Medical College, New York, NY, United States; ^2^Brain and Mind Research Institute, Weill Cornell Medical College, New York, NY, United States

**Keywords:** BDNF, myocyte, cardiovascular system, TrkB, vascular injury, myocardial infarction

## Abstract

The actions of BDNF (Brain-derived Neurotrophic Factor) in regulating neuronal development and modulating synaptic activity have been extensively studied and well established. Equally important roles for this growth factor have been uncovered in the cardiovascular system, through the examination of gene targeted animals to define critical actions in development, and to the unexpected roles of BDNF in modulating the response of the heart and vasculature to injury. Here we review the compartmentally distinct realm of cardiac myocytes, vascular smooth muscle cells, endothelial cells, and hematopoietic cells, focusing upon the actions of BDNF to modulate contractility, migration, neoangiogenesis, apoptosis and survival. These studies indicate that BDNF is an important growth factor which directs the response of the cardiovascular system to acute and chronic injury.

## Introduction

The preponderance of research on the biology of BDNF has focused on its actions upon central and peripheral neurons. Brain-derived neurotrophic factor (BDNF) was first isolated in the 1980s, several decades after the identification of the related factor, nerve growth factor (NGF) (Barde et al., 1982). BDNF plays a critical role in promoting the differentiation of specific classes of neurons, and in modulating synaptic activity [reviewed in Lu et al. (2014)]. BDNF is initially synthesized as a precursor protein, proBDNF, which is cleaved to yield a N-terminal prodomain and a C-terminal protein, mature BDNF. Early studies identified the heart and vasculature as sources of BDNF, based on RNA expression. As other neurotrophins, most notably NGF, are known to critically regulate the development of the peripheral nervous system by providing trophic support in target tissues, BDNF was similarly posited to promote the outgrowth of peripheral neurons to the heart and great vessels during development. Subsequent studies, however, determined that the BDNF receptors (i.e., TrkB – activated by mature BDNF), p75 – activated by the precursor proBDNF or mature BDNF, or sortilin and SorCS2 – activated by proBDNF and the cleaved prodomain were expressed by cardiac myocytes, endothelial cells, vascular smooth muscle cells or hematopoietic cells which contribute to the integrity of the vascular wall. These results suggested that in addition to modulating peripheral neuron development and function (reviewed in Luther and Birren, 2009), BDNF (or its precursor proBDNF, or cleaved prodomain) could exert independent, local actions on the cardiovascular (CV) system. Here we review the expression patterns of BDNF and its receptors in the heart and great vessels, focusing upon roles during cardiac development, defined actions in the adult, and the functions of BDNF following acute and chronic injury as exemplified by acute ischemia, and atherogenesis, respectively. The potential impact on cardiovascular risk of a common human polymorphism in the BDNF prodomain, as well as the relative roles of proBDNF and mature BDNF in vascular injury are described. Finally we compare the effects of BDNF with those of other neurotrophins (NT-3 and NGF/proNGF) in mediating angiogenic actions. Collectively, these studies identify BDNF and its receptors as important potential drug targets to modulate the response of the cardiovasculature to injury.

## BDNF Actions in the Developing Heart

The effects of BDNF on cardiac development were first identified through the study of BDNF null mutant mice, which exhibit early postnatal lethality (Donovan et al., 2000). In addition to defects in peripheral innervation effecting respiratory drive (reviewed in Ogier et al., 2013), BDNF deficient mice exhibit defects in atrial septation, and prominent intramyocardial vessel fragility and hemorrhage, leading to reduction in cardiac ejection fraction as measured by echocardiographic imaging (Donovan et al., 2000). Ultrastructural analysis of hearts from BDNF deficient mice indicate that BDNF promotes the stabilization of cardiac arterioles and capillaries, through effects on TrkB expressing endothelial cells and pericytes/vascular smooth muscle cells (Donovan et al., 2000). Genetic overexpression of BDNF in the developing murine heart results in hypervascularization, and an increase in capillary density (Donovan et al., 2000). These studies suggest that BDNF promotes endothelial, pericyte and vascular smooth muscle cell survival in the heart in late embryogenesis.

**FIGURE 1:**
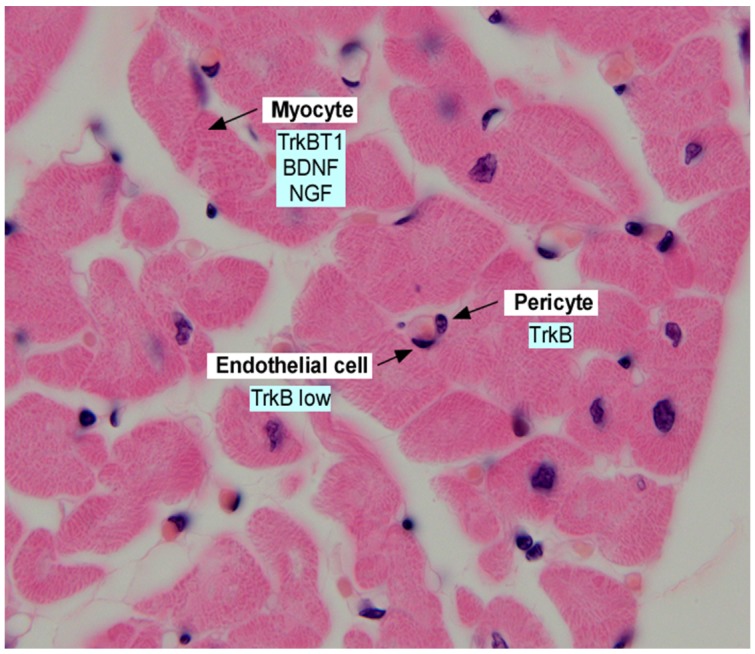
Section of adult human myocardium, stained with hematoxylin and eosin and annotated with expression of neurotrophins and neurotrophin receptors in the indicated cell types. Data was derived from Scarisbrick et al. (1993); Yamamoto et al. (1996), Donovan et al. (2000); Wagner et al. (2005), Anastasia et al. (2014), and Fulgenzi et al. (2015).

Several more recent studies have refined our understanding of BDNF/TrkB in the developing heart through the study of TrkB gene targeted mice. Similar to the deficits noted in the BDNF null animals, embryos lacking TrkB expression demonstrate a reduction in subepicardial vessels in late gestation (Wagner et al., 2005). The mechanism underlying vascular insufficiency in the heart, which leads to late gestational lethality, appears to be due to a reduction in pericytes which contribute to the cardiac microvasculature, and cardiac arterioles (Anastasia et al., 2014). Indeed, tissue specific deletion of TrkB in pericytes/vascular smooth muscle cells resulted in a vascular phenotype of impaired pericyte coverage of cardiac capillaries, however, the vascular defects were not as severe as that observed in TrkB null mice (Anastasia et al., 2014). These results suggest that TrkB may have effects on other vascular cells, such as endothelial cells, in addition to prominent effects on pericytes (Figure 1).

## BDNF Actions in the Adult Heart

While numerous studies have investigated the roles of BDNF on cardiac development, much less is known about the effects of BDNF on adult cardiac function and cardiomyocyte contractility. Although initially described as a receptor tyrosine kinase, the *trkB* locus generates multiple *trkB* isoforms which encode the same extracellular domain, but express different intracellular domains, including a truncated product which lacks tyrosine kinase activity (TrkBT1) (Barbacid, 1994). TrkBTI is the most abundant isoform in the adult murine heart, and is expressed by cardiac myocytes (Fulgenzi et al., 2015), whereas the kinase active TrkB isoform is expressed in the neonatal heart. Furthermore, perfusion of isolated mouse hearts with BDNF induced an increase in cardiac contractile force, with an increased systolic, and decreased diastolic blood pressure (Fulgenzi et al., 2015) and this response was localized to direct effects of BDNF on cardiac myocytes to enhance depolarization-induced Ca^+2^ transients mediated by TrkBTI activation. Indeed, hearts from mice deleted in TrkBT1 fail to exhibit enhanced contractile force in response to BDNF, and these mice develop an adult-onset cardiomyopathy, consistent with the observed expression of the TrkBT1 isoform in adult cardiac myocytes. This study also identified the cardiac myocyte as a significant local source of BDNF, as cardiac myocyte-specific deletion of BDNF induced a similar cardiomyopathy phenotype. While these results do not exclude a role for other neurotrophins, particularly in injury states, they point to the requirement for continued, local synthesis of BDNF to maintain optimal cardiac function. A major, unresolved question relates to how the truncated TrkB isoform (TrkBT1) transduces the inotropic effects of BDNF, which will require additional study.

A concurrent study evaluated the potential impact of the BDNF-mediated activation of the kinase active isoform of TrkB in the adult heart, by genetically deleting this isoform in cardiac myocytes (Feng et al., 2014). Using this approach, deficits in CaMKII activity were observed upon deletion of kinase active TrkB. While deficits in systolic function were observed, these were not significant enough to trigger chamber dilation or reduce ejection fraction. Interestingly, in models of cardiac failure (induced by transverse aortic constriction, or in hearts overexpressing G α q that display cardiac dilation), induction of the truncated isoform (TrkBT1) is observed, suggesting a compensatory mechanism to augment myocyte contractility (Figure 1).

These studies are highly relevant as the field considers the use of BDNF-mimetics to enhance TrkB activation in central neurons for the treatment of mood disorders, regulation of food intake, and neurodegenerative conditions. Novel BDNF mimetics will need to be evaluated for potential effects on the function of the adult cardiovascular system, which could include increased systolic blood pressure. Indeed, delivery of a TrkB agonist antibody to mice induced moderate systolic and diastolic hypertension, an effect which was dose-dependent, and reversible Xu et al. (2010).

## Biosynthesis and Local Secretion of BDNF that Impact Cardiovascular Function

**FIGURE 2:**
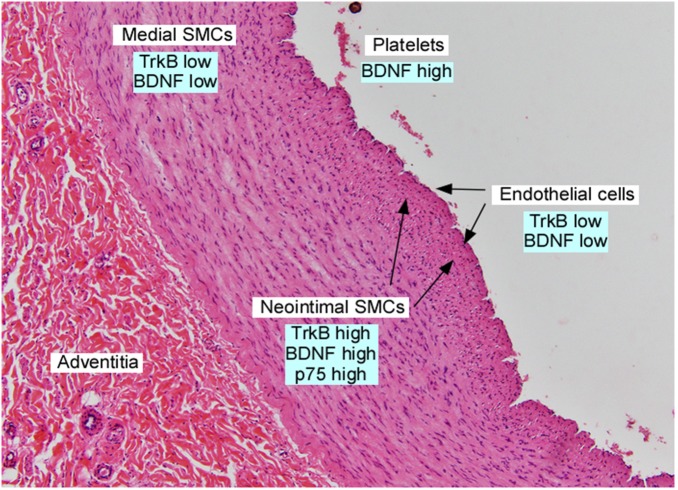
Section of adult human iliac artery, stained with hematoxylin and eosin and annotated with expression of neurotrophins and their receptors in the indicated cell types. Data was derived from Scarisbrick et al. (1993); Donovan et al. (1995), Yamamoto et al. (1996); Kramer (2002), Kraemer et al. (2005), and Chacó-Fernández et al. (2016).

Many studies have evaluated plasma or serum levels of BDNF, in an attempt to correlate circulating blood levels with those available to tissues, such as the brain, myocardium or vessel wall. However, the physiochemical properties of BDNF suggest that following secretion from cells, it acts locally, over short distances. Specifically, BDNF is a highly charged molecule (pKi of ∼9), with a short half-life in the circulation following exogenous delivery, and poor tissue penetration (Harris et al., 2016). While numerous studies have quantified human plasma/serum BDNF levels as an indicator of tissue levels, it is now well recognized that platelets are the primary source of BDNF in human serum (Chacó-Fernández et al., 2016; Figure 2). In humans and other primates, BDNF is synthesized and packaged in the alpha granules of megakaryocytes, the platelet precursor (Chacó-Fernández et al., 2016). Platelets circulate in blood, and following platelet aggregation, BDNF is released with other platelet granule contents. Thus, platelet adhesion to the injured vasculature, which can occur in regions of atherosclerosis or thrombosis, provides high local concentrations of BDNF to the vessel wall. Surprisingly, mouse platelets do not contain significant amounts of BDNF, and the BDNF levels in mouse serum are undetectable (Radka et al., 1996), suggesting that other cell types are a source of BDNF in the murine vessel well. Indeed, murine endothelial cells and vascular smooth muscle cells within the heart and great vessels express BDNF both during development and in adulthood (Yamamoto et al., 1996; Nemoto et al., 1998; Donovan et al., 2000; Nakahashi et al., 2000). However, endothelial cells in other murine organs do not express BDNF at significant levels, suggesting that other growth factors provide pro-survival signals to endothelial and vascular smooth muscle cells in other organs. As noted above, murine cardiac myocytes also express low levels of BDNF, which is required to enhance cardiac myocyte contractility in adulthood (Fulgenzi et al., 2015).

## BDNF Actions on the Vasculature

*In situ* hybridization studies demonstrate that vascular smooth muscle cells in the great vessels and coronary arteries express several neurotrophins, including BDNF, during development. This expression pattern was interpreted as providing trophic support to mediate the outgrowth of developing sympathetic, parasympathetic and cranial nerves (Scarisbrick et al., 1993), with maintained expression of BDNF through adulthood. However, subsequent studies documented that vascular smooth muscle cells in the aorta and coronary arteries express Trk receptors, and neurotrophins induce vascular smooth muscle cell migration (Donovan et al., 1995). Vascular injury, using a rodent aortic balloon injury model, results in rapid and dramatic upregulation of BDNF, TrkB and p75 in the developing neointima (Donovan et al., 1995). This expression pattern is similar to that observed in chronic vascular injury in humans, as specimens of atherosclerotic plaques or atherectomy specimens from restenotic lesions confirm prominent BDNF and TrkB expression primarily in cells co-expressing vascular smooth muscle cell markers (Donovan et al., 1995; Figure 2). TrkB is expressed at high levels by neointimal vascular smooth muscle cells, but not macrophages of the atheromata which develop in the ApoE-/-, cholesterol fed murine model of atherogenesis (Kraemer et al., 2005), suggesting that similar processes are utilized in mice in response to atherogenic stimuli.

To determine whether BDNF/TrkB activation plays a mechanistic role in atheromata formation, TrkB+/- haploinsufficient mice on an ApoE-/- background were studied following exposure to a cholesterol rich diet to enhance atherogenesis (Kraemer et al., 2005). Haplodeficient expression of TrkB reduced vascular lesion size by 30–40%, compared to mice with normal TrkB expression, suggesting that TrkB enhances atheromata formation. Vascular smooth muscle cells expressing TrkB are the most likely cells which contribute to atherogenic plaques, as transplantation of TrkB+/+ bone marrow, as a source of macrophages homing to atheromata, failed to restore vascular lesion development (Scarisbrick et al., 1993). Similarly, conditional deletion of BDNF from endothelial cells, in ApoE-/- mice, failed to alter lesion formation (Norata et al., 2012). Collectively these studies implicate a local paracrine function of increased BDNF expression by vascular smooth muscle cells in regions of chronic injury, that acts locally on neointimal vascular smooth muscle cells, to promote atherogenesis.

While most studies have focused on the actions of BDNF as a TrkB ligand, the rapid induction of p75 by vascular smooth muscle cells (Donovan et al., 1995) following injury prompted further investigation. Proneurotrophins, including proBDNF, can activate p75, in complex with a sortilin family member (sortilin or SorCS2), to mediate apoptosis or acute negative morphological remodeling (Teng et al., 2005; Deinhardt and Chao, 2014). To define a potential role of p75 in vascular injury, p75 null mice were examined in a flow restriction model of carotid injury. Mice deficient in p75 develop vascular lesions which are 3- to 4- fold larger than p75 wild type mice (Kramer, 2002), due to reduced smooth muscle cell apoptosis. These studies suggest that the neurotrophin receptors p75 and TrkB may play opposing roles in regulating the formation of neointimal lesions, with p75 mediating neointimal regression, and TrkB promoting formation of a neointima.

## BDNF and Neovascularization Following Cardiac Injury

**FIGURE 3:**
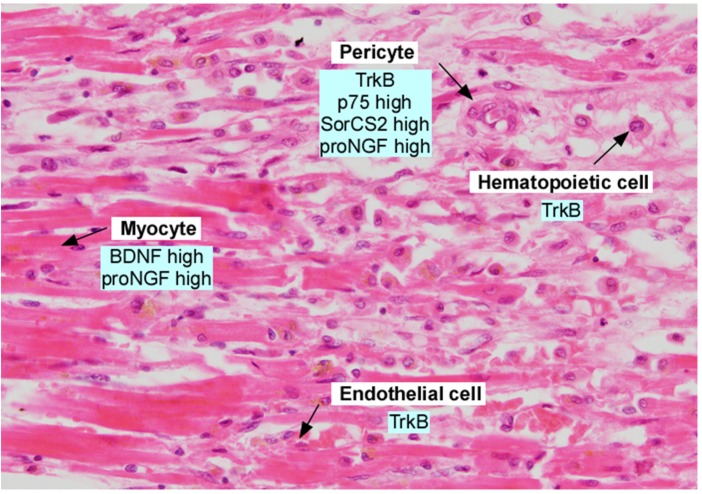
Section of adult human myocardium following myocardial infarction, in the border zone. Section was stained with hematoxylin and eosin and annotated with expression of neurotrophins and neurotrophin receptors. Data was derived from Kermani et al. (2005), Liu et al. (2006), Cai et al. (2006), Okada et al. (2010), Cao et al. (2012), Siao et al. (2012), and Halade et al. (2013).

The above studies describe important actions for BDNF in maintaining cardiac vessel stability in development, in promoting cardiac myocyte contractility in adulthood, as well as playing complex roles following vessel injury. The first study to implicate BDNF as a potential therapeutic ligand described the rapid induction of *bdnf* mRNA and *ngf* mRNA, but not *nt-3* mRNA, following transient cardiac ischemia in the rat (Hiltunen et al., 2001). Hiltunen et al. (2001) noted that *bdnf* mRNA levels doubled in the ischemic region, but this induction lasted only 9 h before returning to baseline. *In situ* analysis suggested that injured myocytes were the source of *bdnf* and this was hypothesized to play a role in innervation or sensory nerve function. More recent studies demonstrate an induction of BDNF and proBDNF protein in the hearts of mice subjected to left coronary artery occlusion, and increased plasma BDNF in patients following acute myocardial infarction(Hang et al., 2015; Figure 3).

To determine whether BDNF could be utilized to promote neovascularization, the effects of BDNF and other neurotrophins to promote cellular recruitment were systematically studied (Kermani et al., 2005). Under non-ischemic conditions, BDNF induced the recruitment of cells expressing markers of endothelial cells, vascular smooth muscle cells, and monocyte/macrophages using a Matrigel model, or a murine ear model in which a durable increase in vascular density was observed in response to BDNF. In an ischemic hindlimb model, delivery of BDNF using adenovirus to provide high levels of BDNF protein expression for one to two weeks, accelerated revascularization to a degree comparable to that obtained using VEGF, the most potent angiogenic factor known to date (Kermani et al., 2005). This effect required expression of TrkB, and involved the specific recruitment of endothelial cells, monocyte/macrophages, and bone marrow derived pro-angiogenic hematopoietic cells. More recent studies suggest, however, that the expression of TrkB isoforms is dynamic in cardiac microvascular cells, with cells from young animals expressing kinase active TrkB, whereas those from aged animals expressing predominantly the truncated TrkB isoform (TrkBT1), leading to a decreased angiogenic potential for BDNF in the hearts of aged animals (Cao et al., 2012).

To directly assess the potential therapeutic actions of BDNF in the ischemic heart, several studies have utilized delivery of mature BDNF protein by intracardiac injection. Conflicting results have been observed, which may reflect species differences (i.e., rats, mice, or dogs), age of animals, or mode and dose of BDNF protein delivered. For example, studies by Hang et al. (2015) evaluated the effects of intramyocardial BDNF injection (1 μg/injection) using rats subjected to acute coronary occlusion and documented beneficial effects at 3 days post infarction, with decreased cardiac myocyte apoptosis, and improved fractional shortening as assessed by echocardiography. In another study (Liu et al., 2006), microsphere mediated delivery of a combination of BDNF (25 μg) and bFGF (100 μg) was more efficacious as compared to bFGF alone (100 μg) in increasing blood flow and fractional shortening, in dogs following experimental myocardial infarction. In contrast to these encouraging results, intramyocardial injection of BDNF (1 μg) into the infarcted region of aged rats (24 months) led to enhanced macrophage infiltration and an increase in infarct size, as compared to vehicle alone (Cai et al., 2006). The differences in outcomes may reflect differences in drug delivery, or recruitment of numerous cell types capable of responding to BDNF in the setting of acute ischemic injury in the heart, and consideration of BDNF as therapy will require further investigation.

Several laboratories have utilized gene targeted mice to elucidate possible functions of BDNF in the setting of acute myocardial injury. Studies comparing BDNF haploinsufficient vs. wild type mice (Halade et al., 2013) noted improved survival and a reduction in negative remodeling of the infarcted region in the BDNF haploinsufficient mice. While a specific mechanism was not identified, BDNF haploinsufficient mice exhibited a reduction in the early influx of neutrophils, and enhancement in the later recruitment of macrophages into the infarcted region. BDNF haploinsufficient mice also exhibited reduced neovascularization, confirming the potentially important role of BDNF in the recruitment of endothelial and hematopoietic cells. Potential confounds of this analysis include the marked change in body habitus of BDNF haploinsufficient mice, with significant increases in body weight due to fat mass, as well as differences in cardiac innervation. To address these potential issues, Okada et al. (2010) used an inducible Cre-Lox system to globally reduce BDNF expression in adulthood (Cai et al., 2006). With this approach, they demonstrated that conditional deletion of BDNF results in a reduced ejection fraction and an increased fibrotic area following myocardial infarction, suggesting that BDNF plays a cardioprotective role. To elucidate a mechanism of BDNF action, the authors studied mice with conditional deletion of TrkB in cardiac myocytes in adulthood. A modest, but statistically significant decrease in ejection fraction and increase in cardiac fibrosis post myocardial infarction were noted (Okada et al., 2010). These results suggest that cardiac myocytes respond to BDNF post-infarction, consistent with the tonic effects of BDNF on normal adult cardiac function described by Feng et al. (2014). However, the most relevant local sources of BDNF post infarct remains to be identified, as conditional deletion of BDNF from cardiac myocytes in adulthood has no effect on cardiac function post MI (Okada et al., 2010). Collectively, these studies suggest that BDNF plays numerous roles to orchestrate the repair mechanisms which are activated following acute myocardial ischemia, with distinct actions on hematopoietic cell recruitment, endothelial cell survival, cardiac myocyte function, and cardiac innervation. Further studies will indeed be required to determine whether BDNF or mimetics which activate TrkB will be useful in the post-infarct state.

## BDNF Val66Met Polymorphism and Cardiovascular Risk

In humans, but not other species, a single nucleotide polymorphism (SNP) in the prodomain region of BDNF Val66Met is observed in approximately 20% of Caucasians, with higher allele frequencies in Asian populations. This polymorphism results in a reduction in the level of mature BDNF which is secreted by cells (Chen et al., 2004). The secreted prodomain, generated following the cleavage of proBDNF to yield mature BDNF and the prodomain, appears to have biological activities (Mizui et al., 2015; Zanin et al., 2017). Specifically the Met66 prodomain differentially activates a receptor complex of p75 and SorCS2 to induce acute negative morphological remodeling of neurons, whereas the Val66 prodomain does not (Anastasia et al., 2013; Giza et al., 2018). While the neuropsychiatric phenotypes of individuals with this Met66 polymorphism have been extensively evaluated (reviewed in Glatt and Lee, 2016), the implications of this SNP on cardiovascular function are less well defined. Evaluation of a large cohort in the Catheterization Genetics study from Duke University implicated the Val/Val genotype with a higher risk than Met allele carriers for clinical cardiovascular events, including more diseased vessels and a lower ejection fraction (Jiang et al., 2017). In a European cohort, the Met allele had a protective role in obesity among healthy individuals. However, no association of the Met allele with coronary artery disease was observed (Sustar et al., 2016). Additional studies will be required to reconcile these findings, and to determine a mechanism by which the Met66 and Val66 alleles impact cardiovascular function.

## Effects of Other Neurotrophins on the Cardiovasculature

*In situ* analysis of mRNA expression has revealed that several neurotrophins are expressed in the heart and great vessels of developing and adult rodents, including *ngf* and *nt-3*, in addition to *bdnf* (Scarisbrick et al., 1993; Hiltunen et al., 1996). Using gene targeted mice, essential roles for NT-3 and its receptor, TrkC were identified in cardiac septation and great vessel formation (Donovan et al., 1996; Tessarollo et al., 1997) with subsequent studies defining NT-3 actions on both developing cardiac myocytes (Lin et al., 2000) and cardiac neural crest (Youn et al., 2003). NT-3 and TrkC are upregulated following aortic injury (Donovan et al., 1995), and direct actions of NT-3 in inducing neovascularization in the post-ischemic state have been identified more recently (Cristofaro et al., 2010).

Nerve growth factor has been well studied for its role in modulating cardiac function, particularly in the setting of injury. Cardiac myocytes express NGF, which functions to establish cardiac sympathetic innervation during development; indeed, the classic experiments of Levi-Montalcini documented the dependence stellate ganglion sympathetic neurons on NGF (Levi-Montalcini and Booker, 1960). In the setting of cardiac ischemia in rats, *ngf* mRNA increases in the ischemic region, with elevated levels in pericytes in the border zone (Hiltunen et al., 2001). Interestingly, *ngf* mRNA levels remain elevated for ∼5 days. Using adenoviral mediated delivery of NGF to the peri-infarct area, increased capillary density and improved blood flow were observed in mice (Meloni et al., 2010), and other studies have demonstrated in non-ishemic models that NGF promote the formation of endothelial cell tubes (Lazarovici et al., 2018). In contrast to these proangiogenic actions, Siao et al. (2012) describe a deleterious function of the precursor form of NGF, or proNGF. ProNGF is a cytokine which utilizes p75 and SorCS2 or sortilin co-receptors to induce apoptosis or acute negative remodeling of cells following injury (Hempstead, 2014). ProNGF is induced following cardiac ischemia in rodents and humans (Siao et al., 2012). ProNGF targets pericytes, to induce pericyte retraction, and promotes microvascular dysfunction through activation of p75 and SorCS2 (Siao et al., 2012). These studies illustrate the distinct effects of proNGF vs. mature NGF, and suggest that these isoforms may be considered as distinct therapeutic targets.

These studies reveal unexpected, and important roles for BDNF, and other neurotrophic factors in the regulation of the development of the heart and great vessels, in the maintenance of adult cardiac function, and complex actions following vascular injury. A key feature of this research is that BDNF plays important roles in this organ system, and that strategies to manipulate BDNF levels in the central nervous system may have unintended consequences in cardiovascular function. The protective role of BDNF in maintaining endothelial, pericyte and cardiac myocyte function and survival provide potential therapeutic strategies to promote neoangiogenesis and stabilization of damaged myocardium following ischemic injury. However, preclinical studies reveal that BDNF can induce the recruitment of multiple hematopoietic cell types to regions of ischemic cardiac injury, and further extensive investigation will be required for the optimal design and delivery of BDNF or mimetics to promote neoangiogenesis. Concurrently, additional studies of the role of BDNF isoforms on acute and chronic vascular injury is warranted, to better understand the interplay of BDNF with other growth factors in atherogenesis.

## Author Contributions

All authors listed have made a substantial, direct and intellectual contribution to the work, and approved it for publication.

## Conflict of Interest Statement

The author BH declares that she received funding from UCB Pharma for unrelated studies. The funder played no role in the study design, the collection, analysis or interpretation of data, the writing of this paper or the decision to submit it for publication. All authors declare no conflict of interest.
